# High Prevalence of Lower Extremity Peripheral Artery Disease in Type 2 Diabetes Patients with Proliferative Diabetic Retinopathy

**DOI:** 10.1371/journal.pone.0122022

**Published:** 2015-03-30

**Authors:** Yi-Wen Chen, Ying-Ying Wang, Dong Zhao, Cai-Guo Yu, Zhong Xin, Xi Cao, Jing Shi, Guang-Ran Yang, Ming-Xia Yuan, Jin-Kui Yang

**Affiliations:** 1 Department of Endocrinology, Beijing Tongren Hospital, Capital Medical University, Beijing, China; 2 Department of Geriatrics, Beijing Tongren Hospital, Capital Medical University, Beijing, China; 3 Department of Endocrinology, Beijing Tongzhou District Luhe Hospital, Capital Medical University, Beijing, China; 4 Beijing Key Laboratory of Diabetes Research and Care, Beijing, China; University of Leipzig, GERMANY

## Abstract

Little is known about the relationship between lower extremity peripheral arterial disease (PAD) and proliferative diabetic retinopathy (PDR) in type 2 diabetes (T2D). Here, we explored the relationship between sight-threatening PDR and PAD. We screened for diabetic retinopathy (DR) and PAD in hospitalized patients with T2D. Patients with a diabetic duration of more than 10 years, HbA1c ≥7.5%, eGFR ≥60mL/min/1.73m2 and with PDR or with no diabetic retinopathy (NDR) were eligible for this cross-sectional study. Severities of DR were graded by digital retinal photographs according to the Early Treatment Diabetic Retinopathy Study (ETDRS) scale. We assessed PAD by measuring Ankle Brachial Index (ABI), Toe Brachial Index (TBI) and Doppler ultrasound. Statistical analyses were performed using SPSS 17.0 software. Of the 1544 patients, 169 patients with extreme eye (57 PDR and 112 NDR) phenotypes met the inclusion criteria. Patients with PDR had a significantly higher proportion of low ABI (≤0.99) and high ABI (≥1.3) than patients with NDR (28.1% and 15.8% vs. 14.3% and 6.2% respectively, P<0.05). PDR patients also had lower TBI than NDR patients (0.56±0.09 vs. 0.61±0.08, P<0.01). The proportion of patients with abnormal duplex ultrasound was higher in PDR than in NDR (21.1% vs. 9.8%, P<0.001). This showed that PDR associated with PAD could be defined in multiple ways: abnormal ABI (≤0.9) (OR = 3.61, 95% CI: 1.15–11.26), abnormal TBI (OR = 2.84, 95% CI: 1.19–6.64), abnormal duplex (OR = 3.28, 95% CI: 1.00–10.71), and critical limb ischemia (OR = 5.52, 95% CI: 2.14–14.26). Moreover, PDR was a stronger independent correlation factor for PAD than a diabetic duration of 10 years. In conclusion, PAD is more common in PDR than in NDR. It implies that PDR and PAD are mostly concomitant in T2D. We should focus on screening PAD in patients with PDR in clinical practice.

## Introduction

Type 2 Diabetes (T2D) often entails micro- and macrovasclar complications. Peripheral arterial disease (PAD) is characterized by reduced blood flow to the lower extremities, which may ultimately require amputation, and has been associated with increased risk of coronary artery disease or stroke. Early diagnosis of PAD may allow for earlier treatment, which could help to prevent or postpone complications associated with PAD. T2D patients with PAD are at increased risk of morbidity and mortality from cardiovascular diseases. Furthermore, PAD is an important risk factor of diabetic foot, and is also one of the major reasons for amputation [1, 2]. It is therefore an urgent task know how to find PAD at an early stage.

Diabetic retinopathy (DR), a microvascular complication, which can be screened easily by digital retinal photographs at an early stage, is now the leading cause of blindness in working-age adults [3]. Generally, DR progresses from non-proliferative diabetic retinopathy (NPDR) to proliferative diabetic retinopathy (PDR). However, diabetes does not necessarily progress to PDR in every patient [4, 5]. The incidence of PDR increases as diabetic duration increases from 0 to 15 years. After 15 years, the incidence of developing PDR remains stable [4, 6]. Many studies have shown that DR was associated with cardiovascular disease (CVD) [7, 8]. Moreover, PDR was more strongly associated with CVD than was NPDR. [8, 9]. PAD has been considered to be one of the subclinical cardiovascular diseases, which implies a possible link between lower extremity PAD and PDR.

To date, there have been few studies about the association of lower extremity PAD with PDR. There were controversial results about the association between DR and PAD defined only by low index of ABI [10, 11]. In this study, we analyze the relationship between sight-threatening PDR and lower extremity PAD assessed by three measures and find whether screening of diabetic retinopathy by digital retinal photographs, which has been used as a routine examination for T2D patients, can be used to predict lower extremity PAD.

## Materials and Methods

### Study population

**Fig 1:**
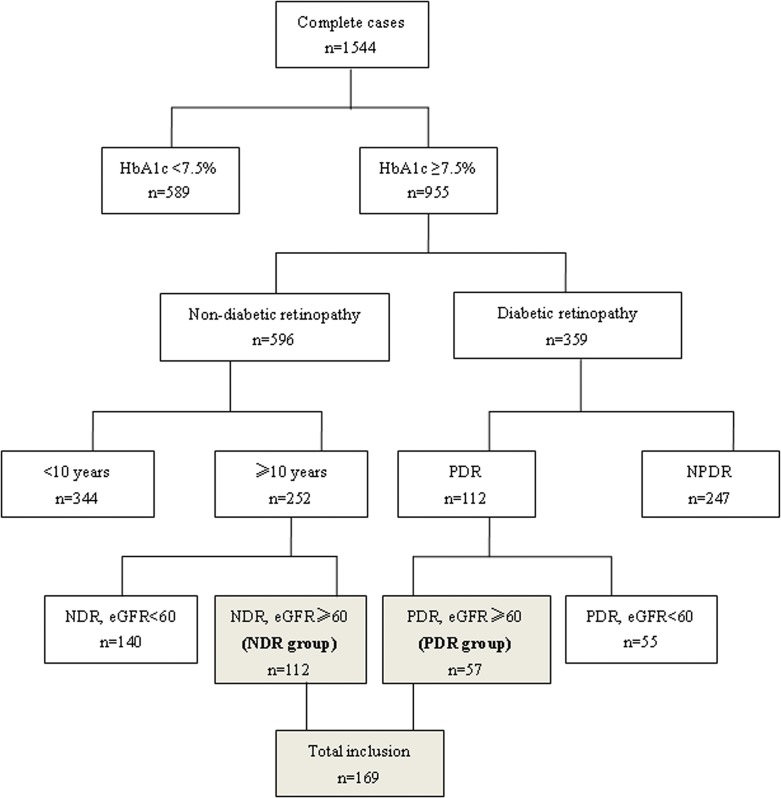
Inclusion and exclusion flow chart.

This is an extreme eye phenotype case-control study. We screened 1544 consecutive patients with T2D who were admitted to the Department of Endocrinology, Beijing Tongren Hospital, Capital Medical University. Diagnosis of T2D was based on the World Health Organization (WHO) criteria [12]. Patients with HbA1c ≥7.5%, eGFR ≥60mL/min/1.73m^2^ and with sight-threatening PDR were assigned as the PDR group. Those patients with diabetic duration of at least 10 years, HbA1c ≥7.5%, estimated glomerular filtration rate (eGFR) ≥60mL/min/1.73m^2^ and with no diabetic retinopathy (NDR) were assigned as the NDR group (See Fig 1). We analyzed the relationship between PDR and lower extremity PAD as assessed by three measures: ABI, Toe Brachial Index (TBI) and Doppler ultrasound.

### Ethics statement

The Medical Ethics Committee of the Beijing Tongren Hospital had approved the study protocol and all participants had provided written informed consent to participate this study.

### Clinical and biochemical parameters

Medical histories studied included history of diabetes, diabetes duration, prior coronary heart disease (CHD, which was defined as history of old myocardial infarction; proven by coronary artery stenosis ≥50% by coronary artery angiography or computed tomography cardiac scanning; past coronary artery bypass grafting and percutaneous coronary intervention). Blood pressure (BP) was measured 3 times with the participant in the seated position, and the average of the last two measurements was adopted. Diagnosis of hypertension was based on meeting any of three criteria: systolic blood pressure (SBP) of ≥140 mmHg, diastolic blood pressure (DBP) of ≥90 mmHg or current use of antihypertensive drugs. We defined SBP <130 mmHg as the target BP in this population. Smoking status, height and weight were recorded, and body mass index (BMI) was calculated. Laboratory parameters including serum creatinine, total cholesterol (TC), triglycerides (TG), HDL cholesterol, LDL cholesterol concentrations and HbA1c were measured. GFR was estimated by using the modified MDRD formula for Chinese patients [13]: eGFR (mL/min/1.73m^2^) = 175 × Scr^−1.234^ × age^−0.179^ × 0.79 (if female). Urinary albumin excretion rate (UAER) was obtained by an 8-hour overnight urine collection.

### Assessment of retinopathy

Two 45° color digital images of the retina, centered at the optic disc and macula, were obtained from both eyes of each participant by an experienced and trained technologist using a TopconTRC-NW7SF fundus camera (Topcon, Tokyo, Japan) ophthalmic digital imaging system. The photographs were graded for retinopathy by the two qualified ophthalmologists from the Eye Center of Capital Medical University, Beijing Tongren Hospital according to the international clinical diabetic retinopathy severity scale [14]. NDR: No microvascular lesions; PDR: Neovascularization of optic disc (NVD) or elsewhere (NVE), preretinal hemorrhage, or vitreous hemorrhage; high-risk characteristics are mild NVD with vitreous hemorrhage, moderate-to-severe NVD with or without vitreous hemorrhage, or moderate NVE with vitreous hemorrhage.

### Assessment of lower extremity PAD

#### ABI and Toe Brachial Index (TBI)

The ABI and TBI were measured using a device (non-invasive vascular screening device VP-2000, Omron, Kyoto, Japan) that allows simultaneous systolic blood pressure measurements from both the upper and lower extremities. In this study, ABI was classified into 3 grades: low ABI (ABI of ≤0.99), normal ABI (ABI 1.0–1.3), and high ABI (>1.3) [15–17]. We defined abnormal ABI as ABI ≤0.9 to diagnose PAD (see Table 1). TBI of <0.6 was defined as “abnormal” in this study [18].

**Table 1:** Subject characteristics by retinopathy category

	Total	NDR	PDR	P
**n**	169	112	57	
**Gender (male/female)**	84/85	57/55	27/30	0.67†
**Age (years)**	58.8±9.6	60.0±9.4	56.6±9.7	0.03
**BMI (kg/m** ^**2**^ **)**	25.3±3.4	25.2±3.26	25.4±3.66	0.64
**Duration of T2D (years)**	13 (10, 18)	12.5 (10, 16.75)	13 (10, 20)	0.99*
**Smoker (%)**	33.1	33.9	31.6	0.86†
**History of hypertension (%)**	49.1	44.6	57.9	0.10†
**History of CHD (%)**	18.9	15.2	26.3	0.08†
**SBP**	128.5±13.9	127.4±13.5	130.3±14.5	0.12
**DBP**	77.0±8.3	76.3±6.9	78.4±8.8	0.12
**FBG (mmol/L)**	8.3 (6.3, 10.0)	8.16 (6.33, 9.25)	8.83 (6.21, 11.21)	0.26*
**A1C (%)**	9.4 (8.2, 10.3)	9.3 (8.23, 10.20)	9.5 (8.10, 10.6)	0.43*
**TC (mmol/L)**	4.8±1.0	4.79±0.97	4.95±1.09	0.33
**TG (mmol/L)** †	1.56 (1.14, 2.26)	1.56 (1.19, 2.34)	1.58 (1.10,2.18)	0.65*
**LDL-C (mmol/L)**	3.11±0.84	3.07±0.80	3.21±0.90	0.30
**HDL-C (mmol/L)**	1.14±0.31	1.14±0.33	1.14±0.26	0.97
**eGFR**	92.1±18.8	91.6±18.1	93.0±20.3	0.66
**UAER**	11.05(5.38, 44.46)	7.34 (4.62, 16.4)	44.28(12.1, 270.42)	<0.001*

BMI, body mass index; A1C: HbA1c; FBG; eGFR,;TC, total cholesterol; TG, triglycerides; LDL-C, low-density lipoprotein cholesterol; HDL-C, high-density lipoprotein; UAER, urinary albumin excretion rate; Student’s t-test

*Mann–Whitney rank test

† Pearson chi-square test. Mean ± SD ormedian (P_25_, P_75_).

#### Doppler ultrasound in the lower extremities

We measured lower limb arteries using B mode and color Doppler (GE company, LogiqE9; 9L linear array ultrasound probe, frequency: 8.4–9MHz). The lower limb arterial segments selected for testing in the anatomical grading system adopted by this study included: (a) iliac; (b) femoropopliteal; (c) posterior tibial; (d) peroneal; and (e) anterior tibial and dorsalis pedis arteries. We used evaluating observation indices: arterial diameter, peak systolic flow velocities (PSV), vascular wall echo, intima-media thickness (IMT), plaque, and stenosis [15]. Three grades were defined: “normal” (PSV <150cm/s or vascular intimal smooth); “borderline” (intimal thickening or plaque or PSV 150–200cm/s); and “abnormal” (artery stenosis >50% or occlusion or PSV >200cm/s).

#### Definition of critical limb ischemia

By modifying the criteria of Critical Leg Ischemia Prevention Study (CLIPS), we defined critical limb ischemia as follows: One ABI <0.85, or one TBI<0.6 when ABI>1.4, or absent flow as measured by color Doppler ultrasound [19].

### Statistical analyses

For the continuous variables with a normal distribution, mean±SD was reported and a Student’s t-test was used to compare the groups. For the discrete variables or the continuous variables without a normal distribution, the median (P25–P75) was reported, and a Mann-Whitney rank test was used to examine the differences between the groups. For the nominal variables, counts and percentages were reported and a Pearson chi-squared test was used to test the difference in percentages between the groups. Binary logistic regression analysis was used to calculate the likelihood of abnormal ABI, abnormal TBI, abnormal duplex ultrasound, and critical lower limb ischemia to extract the independent influence factors with OR (Exp (B)) and 95% CI of OR. A P-value of less than 0.05 was considered statistically significant. Statistical analyses were performed using SPSS 17.0 software.

## Results

### Demography of the participants

In the study population registry of 1544 patients, 169 (57 PDR and 112 NDR) met the inclusion criteria (Fig 1). Patients in the PDR group had a lower mean age and higher UAER levels compared with patients with NDR (P<0.05) (Table 1). Patients in the PDR and NDR groups showed no significant differences in gender, BMI, SBP, duration of diabetes, history of smoking, hypertension, CHD or biochemical parameters (HbA1c, FBG, TC, TG, LDL, HDL, and eGFR).

There were no significant differences in gender, BMI, SBP, duration of diabetes, history of smoking, hypertension, CHD or biochemical parameters (HbA1c, FBG, TC, TG, LDL, HDL, and eGFR) between the PDR and NDR groups.

### PDR and abnormal ABI

**Fig 2:**
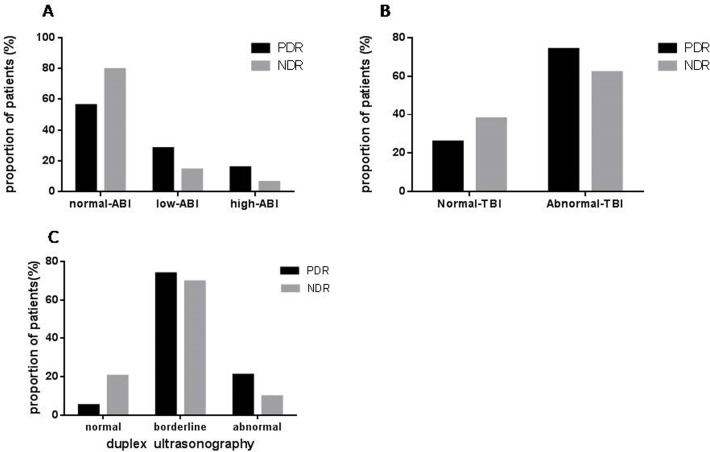
Difference between PDR and NDR in PAD as defined by 3 tools. **Fig 2A: ABI category.** In the PDR patients, 16 (28.1%), 32 (56.1%) and 9 (15.8%) had low, normal and high ABI, respectively. In the NDR patients, 16 (14.3%), 89 (79.5%) and 7 (6.2%) had low, normal and high ABI, respectively. Patients with PDR had higher proportion of abnormal ABI (low ABI or high ABI) than diabetes patients without retinopathy (P<0.05). **Fig 2B:** TBI category. PDR group had higher proportion of abnormal TBI than NDR: 73.7% (42/57) vs. 38.4% (43/112), P<0.01. **Fig 2C: duplex ultrasound category.** In PDR patients, 3 (5.3%), 42 (73.7%) and 12 (21.1%) had normal, borderline, and abnormal duplex respectively. In NDR patients, 23 (20.5%), 78 (69.6%) and 11 (9.8%) had normal, borderline, and abnormal duplex, respectively. The proportion of patients with abnormal duplex ultrasound was significantly higher in the PDR group than in the NDR group (21.1% vs 9.8%, P<0.001).

There was no difference in the ABI value between the PDR and NDR groups (1.06±0.09 vs 1.06±0.08, P = 0.92). The ABI grades between the two groups were significantly different. In PDR patients, 16 (28.1%), 32 (56.1%) and 9 (15.8%) had low, normal and high ABI respectively. In NDR patients, 16 (14.3%), 89 (79.5%) and 7 (6.2%) had low, normal and high ABI respectively (P<0.01, Fig 2A). Patients in the PDR group had a higher proportion of abnormally low or high ABI.

Logistic regression analysis found that together with age (OR = 1.89, 95% CI: 1.09–3.27, P = 0.023), diabetes duration (per 10 years) (OR = 1.60, 95% CI: 1.10–2.34, P = 0.014) and history of hypertension (OR = 3.77, 95% CI: 1.27–11.19, P = 0.017), PDR was an independent predictor for abnormal ABI (ABI ≤0.9) (OR = 3.61, 95% CI: 1.15–11.26, P = 0.027) (See Table 2) after adjusting for CHD, BMI, SBP, UAER, smoking, HbA1c, TC and TG.

**Table 2:** Logistic regression model with the dependent variable as abnormal ABI, abnormal TBI, abnormal duplex, and critical limb ischemia respectively.

	Independent variables	OR	CI	P
**ABI**	Age (per 10 years)	1.89	1.09–3.27	0.023
	duration of diabetes (per 10 years)	1.60	1.10–2.34	0.014
	Hypertension (yes/no)	3.77	1.27–11.19	0.017
	SBP ≥130 mmHg	1.82	0.65–5.09	0.254
	PDR (yes/no)	3.61	1.15–11.26	0.027
**TBI**	Age (per 10 years)	1.31	0.88–1.97	0.189
	duration of diabetes (per 10 years)	1.10	0.82–1.48	0.517
	Hypertension (yes/no)	2.03	0.97–4.24	0.060
	SBP ≥130 mmHg	2.33	1.10–4.92	0.027
	PDR (yes/no)	2.84	1.19–6.74	0.018
**Duplex**	Age (per 10 years)	1.89	1.02–3.48	0.042
	duration of diabetes (per 10 years)	1.41	0.96–2.07	0.076
	Hypertension (yes/no)	2.06	0.66–6.45	0.212
	SBP ≥130 mmHg	1.10	0.37–3.23	0.865
	PDR (yes/no)	3.28	1.00–10.71	0.049
**Critical limb ischemia**	Age (per 10 years)	1.56	1.00–2.44	0.051
	duration of diabetes (per 10 years)	1.24	0.90–1.69	0.185
	Hypertension (yes/no)	1.53	0.68–3.46	0.309
	SBP ≥130 mmHg	1.18	0.51–2.72	0.695
	PDR (yes/no)	5.52	2.14–14.26	<0.001

It was adjusted for the variables shown above as well as duration of diabetes, BMI, UAER, smoking, HbA1c, total cholesterol and triglycerides.

### PDR and abnormal TBI

Our data showed that TBI was lower in PDR patients than in NDR (0.56±0.09 vs 0.61±0.08, P<0.01). The distribution pattern of the TBI grades between the two groups was significantly different. Patients in the PDR group had a higher proportion of abnormal TBI than patients in the NDR group (73.7% vs 38.4%, P<0.01. Fig 2B).

In a logistic regression analysis, PDR was significantly associated with TBI (OR = 2.84, 95% CI: 1.19–6.64, P = 0.018) (Table 2), as was SBP (≥130 mmHg) (OR = 2.33, 95% CI1.10–4.92, P = 0.027) after adjustment for age, duration of diabetes, CHD, BMI, UAER, smoking, history of hypertension, HbA1c, TC, and TG. It showed that PDR remained an independent risk factor in PAD (as assessed by TBI).

### PDR and abnormal duplex

In PDR patients, 3 (5.3%), 42 (73.7%) and 12 (21.1%) had normal, borderline, and abnormal duplex, respectively, while in NDR patients, 23 (20.5%), 78 (69.6%) and 11 (9.8%) had normal, borderline, and abnormal duplex, respectively. The distribution pattern of the duplex findings between the two groups was significantly different (P<0.001) (Fig 2C). PDR patients had higher proportion of borderline or abnormal Duplex ultrasound.

Logistic regression analysis showed that PDR (OR = 3.28, 95% CI1.00–10.71, P = 0.049) was significantly associated with abnormal duplex ultrasound as well as age (OR = 1.89, 95% CI1.02–3.48, P = 0.042) (Table 2). PDR individuals were over threefold more likely to have PAD (assessed by duplex ultrasound) than NDR, even after adjustment for other factors (history of hypertension, history of CHD, BMI, SBP (≥130 mmHg), UAER, smoking, HbA1c, TC, TG).

### PDR and critical limb ischemia

After adjustment for age, history of CHD, history of hypertension, duration of diabetes, SBP (≥130 mmHg), BMI, UAER, smoking, HbA1c, TC, TG, we determined only PDR was significantly associated with critical limb ischemia (OR = 5.52, 95% CI: 2.14–14.26, P<0.001) (Table 2). The risk of critical limb ischemia increased 5.52-fold when PDR was present.

## Discussion

Our data showed there was a significant relationship between PDR and PAD as assessed by ABI, TBI, and duplex ultrasonography. Our data demonstrated that patients with PDR were more likely than patients with NDR to have PAD. Furthermore, a higher proportion of PDR patients had either low or high ABI compared with NDR patients. We also found that PDR was an independent correlation factor of abnormal ABI (≤0.9), which is consistent with previous reports [11]. Few studies analyzed the relationship between PDR and PAD as assessed by TBI. Only one prior study found that TBI is strongly associated with albuminuria in patients with T2D [20]. In this study, the mean TBI value in the PDR patients was lower than in the NDR patients.

Duplex ultrasonography has gradually become a first-line tool for PAD to provide an accurate assessment of lower extremity PAD [21]. We verified the association between PDR and PAD defined by duplex. However, previous relevant studies between PDR and PAD have not used duplex ultrasonography to assess PAD.

ABI, TBI, and duplex ultrasonography are simple, non-invasive ways to assess PAD. Prior studies have mostly used just a single index to assess PAD. However, each indicator has its own advantages and disadvantages. ABI is a quick, cost-effective method to screen for lower extremity PAD but might be less accurate in diabetes patients with non-compressible arteries [21, 22]. TBI, which can be used in subjects with non-compressible arteries, is mostly used to assess toe perfusion when small vessel artery disease is suspected [15, 21]. Limitations of duplex imaging include obesity, joint contracture, and arterial wall calcification [21]. Thus, we defined critical limb ischemia with strict criteria [19]. In order to increase the accuracy of PAD diagnosis, we combined 3 tools to define PAD presenting as critical limb ischemia. Our current study demonstrates clearly that PDR was not only associated with vessel stiffness as assessed by ABI and TBI, but also with atherosclerosis as evaluated by duplex imaging.

Mounting evidence points to the relationship between the microvascular and macrovascular complications of T2D. The association between DR and CVD has been well established. In particular, most studies have shown that PDR was more strongly correlated with CVD when compared with NPDR [23, 24]. A recent study in the ACCORD trial has also shown that the severity of retinopathy is one of the determinants of incident CVD outcomes [25]. PAD is a subclinical CVD. However, most studies focus on the association between PAD and nephropathy rather than between PAD and retinopathy. We observed an independent association between PDR and PAD. Although some studies did not show the association between DR and PAD, they did not grade the severity of DR [10, 26]. Not all diabetes patients will develop DR [27]. The main pathological change in NPDR is increased vascular permeability, whereas in PDR, the main change is neoangiogenesis. Therefore, it is inferred that the principal pathogenetic mechanisms for NPDR and PDR are different [28].

The present study implies that T2D patients with PDR have a high prevalence of PAD. Several explanations may account for the link between PDR and PAD. First, neovascularization (retinal angiogenesis) is a key hallmark of PDR [29], and angiogenesis is also a common feature observed in advanced atherosclerotic lesions. Second, PDR is strongly related to the degree of several independent determinants of PAD, including hyperglycemia, blood pressure, dyslipidemia, albuminuria, and other abnormalities. In addition, many studies found that some patients with diabetes develop severe retinopathy or PAD earlier and more aggregative than others, which were independent of glycemic control and measured environmental factors [30–34].

Our study is a single center, case-control and cross-sectional study. We cannot exclude a systematic bias based on the mode of the selection of our patients. Therefore, the results obtained here cannot be extended to all diabetic subjects. These novel findings need confirmation in large cohorts and in prospective studies. We did not use peripheral arteriography to diagnose PAD because it is invasive and expensive. We also did not select more accurate noninvasive measures such as Magnetic Resonance Imaging (MRI) and computed tomography (CT), which are costly and unsuitable for clinical screening.

## Conclusions

In conclusion, PDR is closely associated with PAD as evaluated by different measurements in T2D. It is inferred that PAD and PDR are concomitant morbidity in patients with diabetes. PDR can be used as a ‘window’ for screening or finding PAD and other macrovascular complications in T2D. We should pay more attention to screening PAD in patients with PDR in order to identify and prevent systematic vascular diseases earlier.
